# Efficient co-production of EPA and DHA by *Schizochytrium* sp. via regulation of the polyketide synthase pathway

**DOI:** 10.1038/s42003-022-04334-4

**Published:** 2022-12-09

**Authors:** Wang Ma, Mengzhen Liu, Zixu Zhang, Yingshuang Xu, Pengwei Huang, Dongsheng Guo, Xiaoman Sun, He Huang

**Affiliations:** 1 School of Food Science and Pharmaceutical Engineering, Nanjing Normal University, 2 Xuelin Road, Qixia District, Nanjing, China; 2 College of Life Sciences, Nanjing Normal University, 2 Xuelin Road, Qixia District, Nanjing, China; 3 College of Biotechnology and Pharmaceutical Engineering, Nanjing Tech University, No. 30 South Puzhu Road, Nanjing, China

**Keywords:** Food microbiology, Microbiology techniques, Metabolomics

## Abstract

Presently, the supply of eicosapentaenoic acid (EPA) and docosahexaenoic acid (DHA) traditionally produced by marine fisheries will be insufficient to meet their market demand in food industry. Thus a sustainable alternative source is urgently required. *Schizochytrium* sp. is an ideal producer of DHA; however, its ability to co-produce DHA and EPA has not yet been proved. Herein, we first described a cobalamin-independent methionine synthase-like (*MetE-like*) complex, which contains independent acyltransferase and 3-ketoacyl synthase domains, independent of the traditional polyketide synthase (PKS) system. When the *MetE-like* complex was activated, the EPA content was increased from 1.26% to 7.63%, which is 6.06-folds higher than that in the inactivated condition. Through lipidomics, we find that EPA is more inclined to be stored as triglyceride. Finally, the EPA production was enhanced from 4.19 to 29.83 (mg/g cell dry weight) using mixed carbon sources, and the final yield reached 2.25 g/L EPA and 9.59 g/L DHA, which means that *Schizochytrium* sp. has great market potential for co-production of EPA and DHA.

## Abstract (web summary)

The MetE like complex is found to be capable of regulating the PKS system to produce EPA in Schizochytrium, with its activation regulating the expression of key enzymes, improving the efficiency of DHA and EPA production.

## Introduction

Very long-chain polyunsaturated fatty acids (VLC-PUFAs), including eicosapentaenoic acid (EPA; C20:5 n-3) and docosahexaenoic acid (DHA; C22:6 n-3), play an important role in reducing the risk of cardiovascular disease and promote brain development^1^. Moreover, EPA and DHA as n-3 supplements can potentially be used as an adjuvant for cardiac issues associated with coronavirus disease 2019 (COVID-19)^2^. Owing to the nutritional value, n-3 supplements were sold $200 million a year in Australia, and sales still increased by 10% per year. The global market size for n-3 supplements was valued at the USA $2.49 billion in 2019 and is expected to increase at a growth rate of 7% from 2020 to 2027^3,4^. Traditionally, EPA and DHA are mainly produced by marine fisheries. However, the traditionally produced EPA and DHA are unable to meet their increasing industrial demand. Therefore, the use of oleaginous microorganisms for the commercial production of EPA and DHA has garnered increasing attention^5,6^.

Thraustochytrids are marine unicellular heterotrophic microorganisms. These organisms have a remarkable ability to accumulate VLC-PUFAs. Based on their life cycle, morphology, ultrastructure, phylogenetic analysis, and biochemical markers^7^, thraustochytrids can be divided into the following 10 genera: *Thraustochytrium*, *Aurantiochytrium*, *Schizochytrium*, *Japonochytrium*, *Parietichytrium*, *Botryochytrium*, *Ulkenia*, *Sicyoidochytrium*, *Monorhizchytrium,* and *Hondaea* genera^8,9^. Almost all thraustochytrids can accumulate DHA. The reported proportion of DHA in total fatty acids (TFAs) is 5.1% in *Parietichytrium* sp. and 54.6% in *Thraustochytrium roseum* ATCC 28210^10,11^. Presently, thraustochytrids are preferred being used in scientific research and DHA commercial production due to their high lipid content (>50% of dry weight) and ease of cultivation. They are preferred for scientific research and the commercial production of DHA^12^. However, achiveing the co-production of EPA and DHA by thraustochytrids remains a challenge. Jiang et al. reported that the proportion of EPA in thraustochytrids did not exceed 1% by comparing the fatty acid composition of five thraustochytrids^13^. Similarly, Fan et al. also obtained only about 0.2% EPA by fermenting *Schizochytrium mangrovei* FB3^14^. To date, two metabolic pathways have been identified in thraustochytrids that synthesize VLC-PUFAs, a conventional aerobic fatty acid elongase/desaturase pathway (ELO/DES) pathway and an anaerobic polyketide synthase (PKS) pathway^15^. Among thraustochytrids, some contain both PKS and ELO/DES pathways^16^. However, most thraustochytrids have only a single PKS or ELO/DES pathway^17^, which can lead to differences in the synthesis of EPA the difference. However, no study has shown which thraustochytrids are the most suitable for the co-production of DHA and EPA.

Nevertheless, thraustochytrids still possess the natural advantage of synthesizing VLC-PUFAs. Therefore, many researchers tried to improve thraustochytrids EPA production by changing the culture conditions or regulating their metabolism. By optimizing the medium composition, *Thraustochytrium* sp. KK17-3 can accumulate EPA exceeds 4.5% of TFAs^18^. Through adaptive laboratory evolution, Sun et al. achieved 3.51% accumulation of EPA by regulating *Schizochytrium* sp.^19^. Still, the effects of altering the culture condition on EPA accumulation by changing the culture conditions were not distinct. Moreover, the use of genetic engineering to modify the fatty acid synthesis pathway of thraustochytrids to synthesize EPA is also a common technique in recent years. For example, the levels of EPA in *Aurantiochytrium limacinum* mh0186 increased 4.6-fold via gene expression of the fatty acid delta 5 desaturase driven by the *Thraustochytrium aureum* ATCC 34304 ubiquitin promoter; however, EPA still accounted for only 2.85% of TFAs^20^. Although the PKS system in thraustochytrids is the main pathway to synthesize VCL-PUFAs, only the DHA/n-6 docosapentaenoic acid (DPA; C22:5 n-6) type PKS gene cluster was isolated from thraustochytrids, whereas EPA or arachidonic acid (ARA;C20:4 n-6) type PKS gene clusters have been identified in many marine bacteria^21,22^. In the bacterial-derived PKS pathway, Orikasa et al.^23^ reported that DHA-*Pfa* B and EPA-*Pfa* B were the key factors determining the final product as DHA or EPA, whereas Dairi et al.^21^ believed that the control mechanism of carbon chain length (C20 or C22) is controlled by the *Pfa* C domain. Based on this, Chen et al. replaced the *Pfa* B-AT domain from *Shewanella* sp. with the *orf* B domain of *Schizochytrium* sp., and the content of EPA in TFAs increased from 1.04% to 3.84%^24^. In addition, to improve the EPA production capacity of thraustochytrids, Wang et al. transferred the EPA-type PKS of *Shewanella japonica* into *Aurantiochytrium* sp. SD116, which led to an increase in EPA from 0.58% to 3.05%^25^. However, the synthesis mechanism of the PKS pathway is yet to be elucidated, and the genetic manipulation tools requires further improvement. Therefore, no breakthrough has occurred in the genetic engineering of thraustochytrids to produce EPA.

In this study, to determine the most potential co-producer of EPA and DHA, we first selected thraustochytrids and analyzed their ability to co-produce EPA and DHA, and *Schizochytrium* sp. was selected for further evaluation (40.35% DHA and 3.57% EPA at 15 °C). Through transcriptomics, we discovered a cobalamin-independent methionine synthase-like (*MetE-like*) complex could modulate the DHA/n-6DPA-type PKS system to produce EPA and verified its function by determining its regulation at the transcriptional level. The *MetE-like* complex can be activated in the absence of cobalamin, which greatly increases the EPA content (from 1.23% to 7.63%), and 9.59 g/L DHA and 2.25 g/L EPA were obtained through fermentation regulation. To the best of our knowledge, this is the first study to report that the endogenous gene *MetE-like* in *Schizochytrium* sp. can help to produce EPA by regulating the PKS system. It makes *Schizochytrium* sp. possible to be an industrial co-producer of EPA and DHA by rational design.

## Results and discussion

### Evaluation of the co-production capacity of thraustochytrids to produce EPA and DHA

**Fig. 1:**
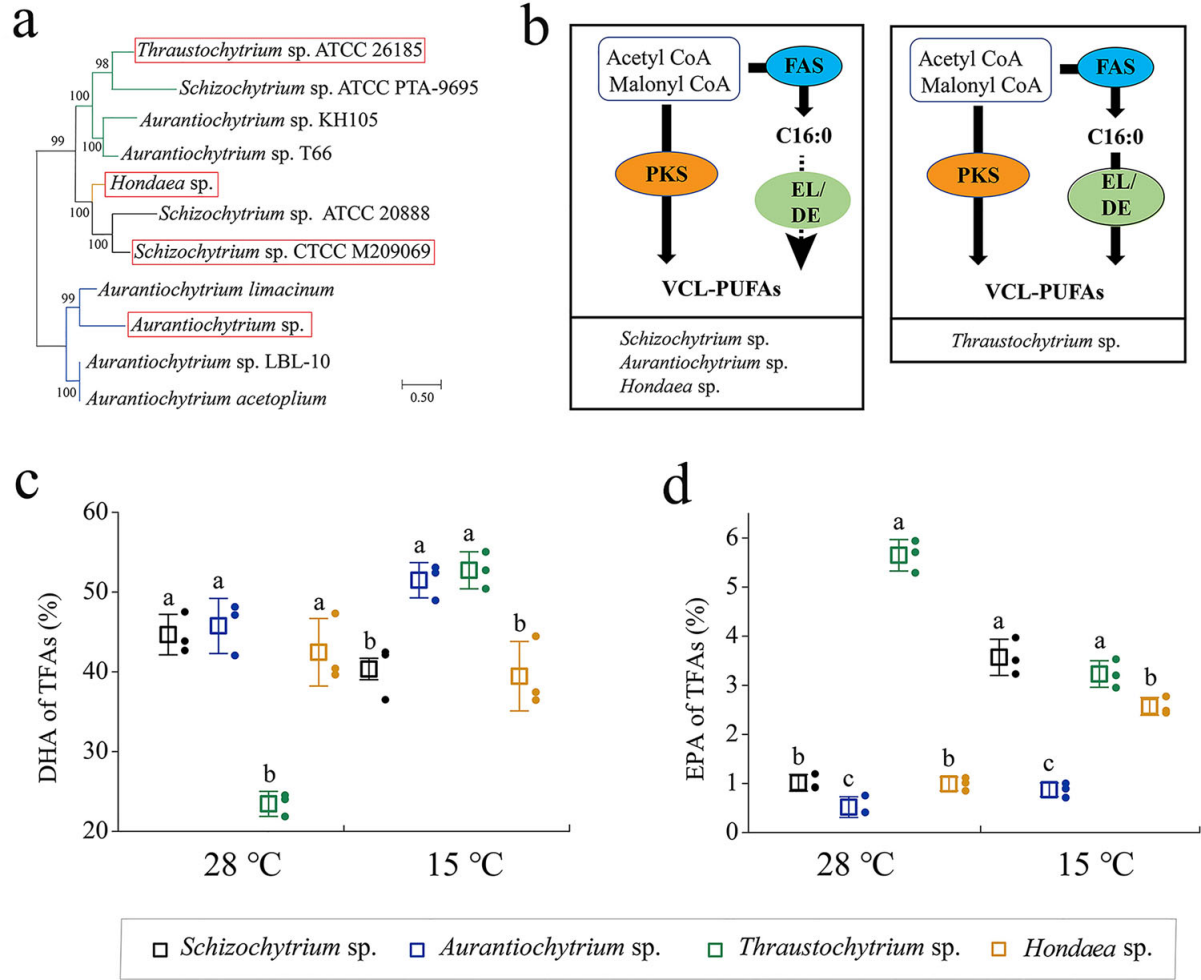
Selection and evaluation of the co-production capacity of different thraustochytrid genera to produce EPA and DHA. **a** Unrooted phylogenetic tree of thraustochytrid genera based on the amino acid sequences of the PKS domain. **b** Schematic diagrams for two types of VCL-PUFAs synthesis systems in thraustochytrids. VCL-PUFAs is synthesized only by PKS pathway (e.g., *Schizochytrium* sp., *Aurantiochytrium* sp., and *Hondaea* sp.), or by both the PKS and EL/DE pathways (e.g., *Thraustochytrium* sp.). **c**, **d** DHA ratio (**c**) and EHA ratio (**d**) of the different thraustochytrid genera under 28 °C and 15 °C. Letters above the bars indicate significant difference (*p* ≤ 0.05), based on one-way analysis of variance (ANOVA) and Tukey’s honestly significant difference (HSD) test. Source data are provided as a Source data file.

The unrooted tree analysis was performed with PKS from thraustochytrids. Even though the fatty acid composition of different thraustochytrid genera differed because of the existence of independent evolution, all PKS pathways are considered to be DHA/n-6 DPA type^21,26^. Finally, we selected *Schizochytrium* sp., *Aurantiochytrium* sp., *Thraustochytrium* sp., and *Hondaea* sp. in different clades from the phylogenetic tree to evaluate the EPA and DHA co-production capacity of the thraustochytrids (Fig. 1a). *Thraustochytrium* sp. can utilize the ELO/DES pathway to produce VCL-PUFAs^16^. However, the ELO/DES pathway of the other three strains was considered incomplete (Fig. 1b). Under the fermentation temperature of 28 °C, *Schizochytrium* sp., *Aurantiochytrium* sp., and *Hondaea* sp. could accumulate more than 40% DHA, which showed excellent DHA synthesis ability, but DHA in *Thraustochytrium* sp. only accounts for 23.45% of TFAs (Fig. 1c). *Thraustochytrium* sp. showed the strongest EPA synthesis ability among the four strains, and the content of EPA was up to 5.65%, which was almost 7 times that of *Schizochytrium* sp. The EPA synthesis ability of *Aurantiochytrium* sp. and *Hondaea* sp. was also far inferior to that of *Thraustochytrium* sp.

Low-temperature conditions are considered to be more conducive to the accumulation of VCL-PUFAs^27^. To further investigate the potential of the four strains to co-produce DHA and EPA, we performed fermentation at 15 °C. Surprisingly, the DHA synthesis ability of *Thraustochytrium* sp. was increased, but its EPA content was only 57.2% of that at 28 °C. Correspondingly, the EPA accumulation capacity of *Schizochytrium* sp. and *Hondaea* sp. was increased, and the final proportion of EPA in *Schizochytrium* sp. was increased by 3.4 times compared with that at 28 °C. Interestingly, the co-production capacity of EPA and DHA in *Schizochytrium* sp. and *Hondaea* sp. was basically the same, whereas the VCL-PUFAs synthesis capacity of *Aurantiochytrium* sp. was less affected by temperature. Therefore, there is an adjustable EPA synthesis pathway in *Schizochytrium* sp. and *Hondaea* sp. Moreover, compared with *Hondaea* sp., *Schizochytrium* sp. has more advantages in biomass and lipid accumulation; therefore, we selected *Schizochytrium* sp. for further evaluation (Supplementary Fig. S1).

### Elucidating the EPA biosynthesis pathway in *Schizochytrium* sp

**Fig. 2:**
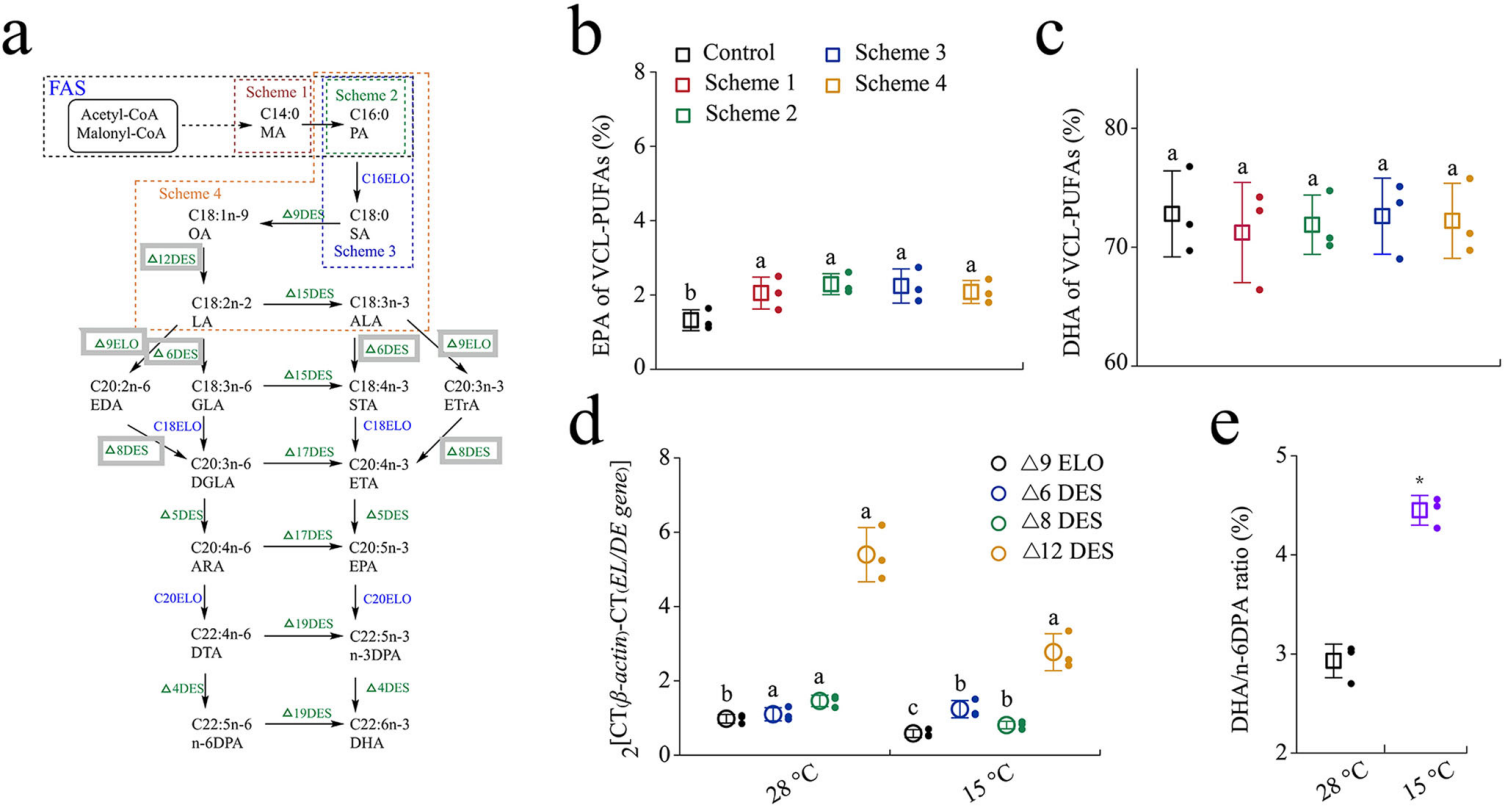
Elucidating the synthetic pathway of VCL-PUFAs in *Schizochytrium* sp. **a** Schematic diagram of the aerobic pathways for VCL-PUFAs biosynthesis. The native EL/DE gene in *Schizochytrium* sp. is indicated with gray solid frame, and the four addition schemes are marked with colored dotted lines. MA myristic acid, PA palmitic acid, STA stearidonic acid, LA linoleic acid, ALA α-linolenic acid, EDA eicosadienoic acid, GLA γ-linolenic acid, STA stearidonic acid, ETrA eicosatrienoic acid, ETA eicosatetraenoic acid, DGLA dihomo-γ-linolenic acid, DTA docosatetraenoic acid. **b**, **c** EPA ratio (**b**) and DHA ratio (**c**) with different addition schemes in *Schizochytrium* sp. **d** Comparison of ELO/DES genes transcript level between 28 °C and 15 °C of *Schizochytrium* sp. (24 h). Letters above the bars indicate significant difference (*p* ≤ 0.05), based on one-way analysis of variance (ANOVA) and Tukey’s honestly significant difference (HSD) test. **e** Comparison of DHA/n-6DPA ratio between the two temperatures. The statistical significances of the final results were analyzed by t-test, ^∗^*p* < 0.05.

In *Schizochytrium* sp., known final products synthesized by the PKS pathway are DHA and n-6DPA, but the synthesis method of EPA has not been elucidated^15,28^. Several genes related to the fatty acid ELO/DES pathway are present in the *Schizochytrium* sp. genome^29^, and this result was also found in *Aurantiochytrium. Limacinum*^30^ and *H. fermentalgiana*^9^. However, no direct evidence that *Schizochytrium* sp. has a complete ELO/DES pathway for EPA synthesis has been found. To confirm the effect of the partial ELO/DES pathway present in *Schizochytrium* sp. on EPA accumulation, a protocol for exogenous addition of ELO/DES substrates was designed, which involved the following four distinct addition schemes (2 g/L): only C14:0, only C16:0, C16:0, and C18:0, and linseed oil (Fig. 2a). In this pathway, specific oxygen-dependent desaturases and elongases catalyzed individual desaturation and elongation steps to synthesize VCL-PUFAs from oleic acid (OA; C18:1 n-9), EPA and ARA as the actual production representatives^31^. In such conditions, schemes 1, 2, and 3 increased EPA synthesis by adding C18:1 precursor fatty acids, whereas scheme 4 contained C18:1 downstream products, which were more favorable for EPA synthesis for the ELO/DES pathway (Supplementary Fig. S2). For EPA, all fermentation schemes increased slightly, which was inconsistent with our assumption that EPA in scheme 4 was not obviously higher than that in other schemes (Fig. 2b). Furthermore, none of the schemes had any effect on DHA synthesis (Fig. 2c). In addition, all the schemes did not affect the growth of *Schizochytrium* sp., which may be related to the low amount of fatty acids we added externally (Supplementary Fig. S3).

Furthermore, we quantified the transcript levels of ELO/DES genes at different fermentation temperatures by reverse transcription (RT)-PCR, since the ability of *Schizochytrium* sp. for accumulating EPA was increased by temperature control (Fig. 1d). Compared with the transcript levels at 28 °C, only the transcript level of △6 DES increased by 22% at 15 °C. However, the transcript levels of △9 ELO, △8 DEO, and △12 DEO all decreased by different magnitudes (Fig. 2d). Moreover, the DHA/n-6 DPA ratio of *Schizochytrium* sp. reached 4.45 at 15 °C, whereas the proportion of DHA did not increase obviously. This indicated a decrease of n-6 DPA (Fig. 2e). Previous studies have reported that DHA and n-6 DPA are characteristic fatty acids of the PKS pathway in *Schizochytrium* sp., and the DHA/n-6 DPA ratio is from 2.6 to 3.5^32,33^. Based on this, *Schizochytrium* sp. can have a regulatory mechanism that can release EPA from the DHA/n-6DPA-type PKS pathway, but the current quantitative data are limited^18,24,25^.

### Identifying a *MetE-like* domain that regulates DHA/n-6 DPA type PKS synthesis of EPA

**Fig. 3:**
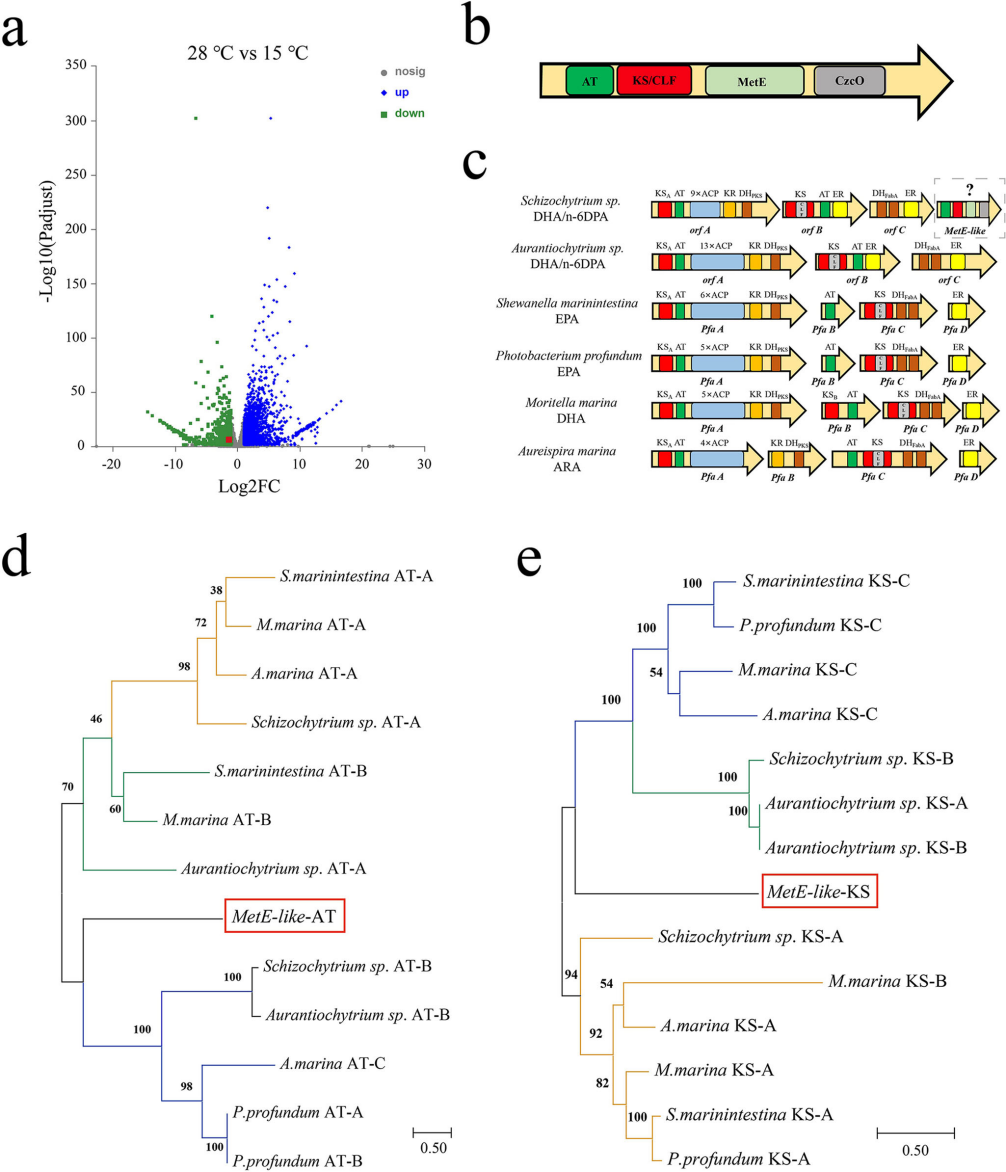
Identifying a *MetE-like* domain relevant to EPA synthesis. **a** Volcano plot of the whole transcriptome. Blue and green dots represent proteins with obviously increased and decreased levels, respectively, gray dots represent genes with similar transcript levels under two growth conditions (*MetE-like* is circled in red). **b** Functional domains included in *MetE-like*. **c** Structure of PKS pathway from microorganisms. DH Dehydratase, KR Ketoacyl-ACP reductase. **d**, **e** Maximum Likelihood phylogenetic analysis was performed with all AT (**d**) and KS (**e**) domain sequences from *Schizochytrium* sp., *Aurantiochytrium* sp., several bacteria and *MetE-like* domain.

To determine how temperature affects fatty acid synthesis in *Schizochytrium* sp., we performed comparative transcriptomics on cells grown under two growth conditions (28 °C and 15 °C). Among all differentially expressed genes, one gene (red rectangle) with both AT and KS domains exhibited obviously transcriptional level upregulation (Fig. 3a). This gene is named *MetE-like*, and it consists of the following four domains: AT domain, KS (or KS/CLF) domain, cobalamin-independent methionine synthase domain (MetE), and flavoprotein domain (CzcO) (Fig. 3b). In fact, the PKS organization can be different in different organisms. As shown in Fig. 3c, unlike the Thraustochytrids-derived PKS system, the ER domain exists independently in bacteria, whereas the AT domain in the EPA-type PKS system also generally exists independently. Besides, free functional domains are present in *Psychroflexus torquis* (ARA/EPA-type PKS), and *Pseudoalteromonas* sp. (EPA-type PKS), which indicates the independent existence of functional domains in the PKS pathway does not affect the function of the PKS system^15^.

To determine the function of AT and KS domains located on *MetE-like*, a maximum likelihood phylogenetic analysis was performed on all AT and KS domain sequences from *Schizochytrium* sp., *Aurantiochytrium* sp., many bacteria, and *MetE-like* domains. The unrooted tree analysis distinctly showed the three main well-supported clades, and AT domain of *MetE*-*like* had high homology with the AT domain of *Pfa* C from *Aureispira marina*, followed by the AT domains of *Pfa* A and *Pfa* B from *Photobacterium profundum* (Fig. 3d). The PKS pathways of *A. marina* and *P. profundum* could synthesize ARA and EPA, respectively. Therefore, the *MetE-like*-AT may participate in the synthesis and release of C20 fatty acids just like the AT domain of *A. marina* and *P. profundium*. In the fatty acid synthesis pathway, thioesterases are required to release free fatty acids from the intermediate metabolites of acyl carrier protein tether. Similarly, the AT domains in PKS of *Schizochytrium* sp. and marine bacteria can catalyze the chain-release reaction of acyl-ACP to form free fatty acids^22^. And, Orikasa et al. reported that the Pfa B-AT domain is the key enzyme in determining the final product of EPA-type PKS and DHA-type PKS^23^. In addition, the KS domain of *MetE-like* had higher homology with the KS domain of *Pfa* C from bacteria (Fig. 3e). In fact, it has been hypothesized that the origin of PKS gene clusters in thraustochytrids is laterally transferred from marine bacteria^34^, which might explain the similarity of *MetE-like-*KS with bacteria-KS. Tang et al.^35^ hypothesized that KS/CLF is the primary determinant of polyketide chain length. However, another study reported that the final length of the carbon chain is determined by both KS_A_ and KS_C_ (KS/CLF), and the KS_A_ domain catalyzes the condensation of C18 to C20. The final elongation step in DHA synthesis is catalyzed by the KS_C_ domain in DHA synthase^21^. Therefore, the KS domain of *MetE-like* may not catalyze the elongation from C20 to C22. Based on this, *MetE-like* can have the function of releasing EPA from the DHA/n-6 DPA-type PKS system.

### Activating the expression of *MetE-like* for enhancing EPA biosynthesis

**Fig. 4:**
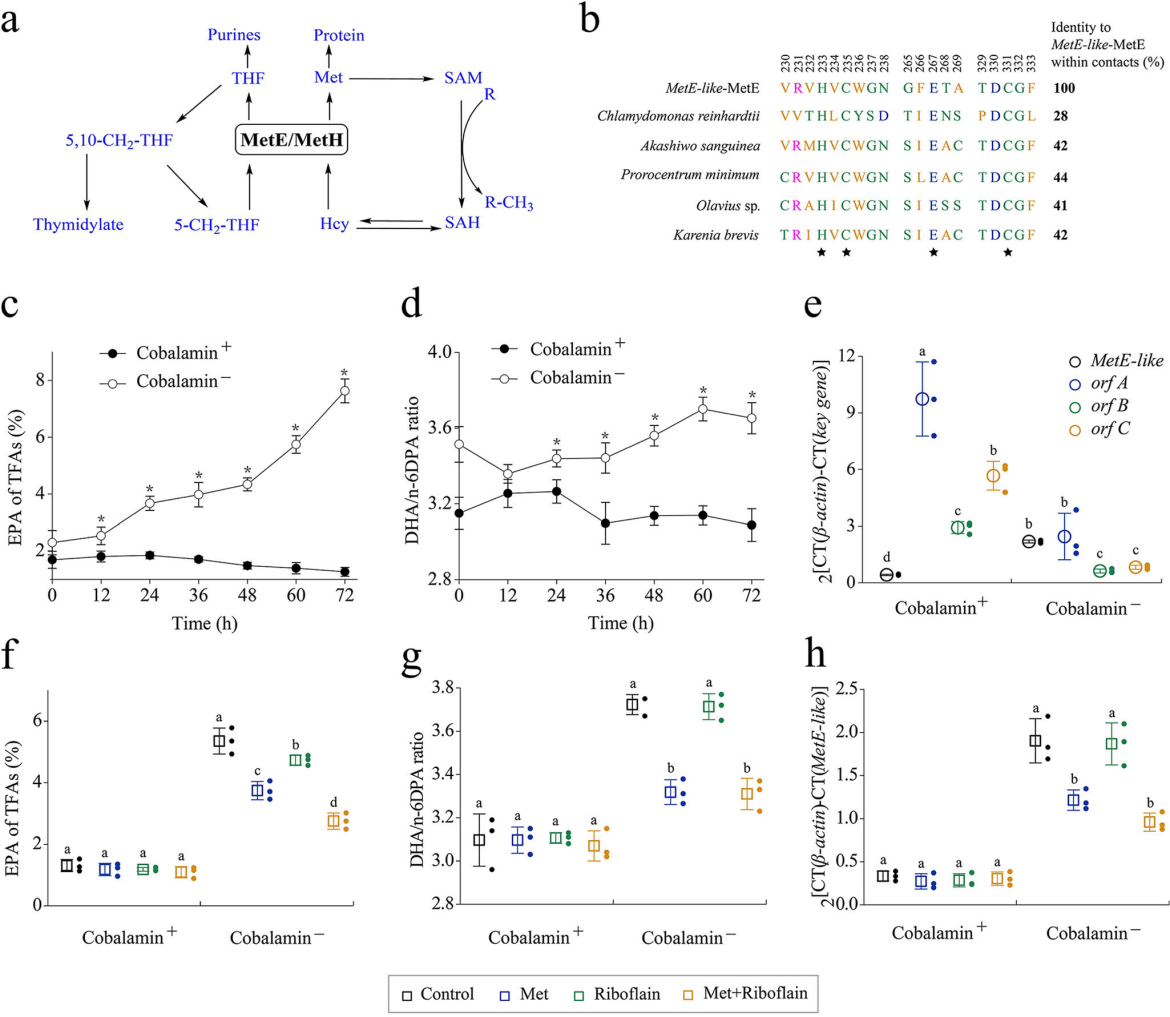
Cobalamin controls the expression level of *MetE-like*. **a** Metabolic map of a portion of the C1 cycle centered around MetE and MetH, with enzyme abbreviations in black, metabolite abbreviations in blue, and arrows depicting enzyme-catalyzed reactions. Hcy Homocysteine, THF tetrahydrofolate, SAH S-adenosine homocysteine, SAM S-adenosyl methionine, 5-CH_2_-THF 5-CH_2_-tetrahydrofolate, 5,10-CH_2_-THF 5,10-CH_2_-tetrahydrofolate. **b** Multiple sequence alignment of MetE protein sequences from eight algae for the region spanning the functionally important conserved residues required for binding Zn^2+^ (indicated by a black star), numerical values on amino acids represent the position of each amino acid in the *MetE-like*-MetE domain. **c**, **d** Time courses of EPA ratio (**c**) and DHA/n-6DPA (**d**) ratio between cobalamin^+^ and cobalamin^−^ of *Schizochytrium* sp. **e** Comparison of PKS genes and *MetE-like* transcript level between cobalamin^+^ and cobalamin^−^ of *Schizochytrium* sp. (60 h). **f**, **g**, **h** Effects of different addition schemes on EPA ratio (**f**), DHA/n-6DPA ratio (**g**), and *MetE-like* transcript level (**h**) between cobalamin^+^ and cobalamin^−^ of *Schizochytrium* sp. (60 h). Letters above the bars indicate significant difference (*p* ≤ 0.05), based on one-way analysis of variance (ANOVA) and Tukey’s honestly significant difference (HSD) test. The statistical significances of the final results were analyzed by t-test, ^∗^*p* < 0.05.

Methionine synthase (MetH) allows the conversion of 5-methyltetrahydrofolate and homocysteine to tetrahydrofolate and methionine (Met)^36^, respectively. It plays a central role in the C1 cycle (Fig. 4a). However, the co-factor cobalamin is required for the catalytic activity of MetH^37^. Unlike MetH, the MetE domain in *MetE-like* can replace MetH to function as MetH in the absence of cobalamin^38^. Sequence similarity searches identified this putative MetE sequence as *MetE-like*. However, it has low homology with other sources of MetE, which can affect the function and activity of *MetE-like*-MetE (Fig. 4b). The C-terminus of MetE contains conserved Zn^2+^-binding residues that are required for catalytic activity^39^. These residues, namely His233, Cys235, Glu267, and Cys330 in *MetE-like*, are conserved in all of the obtained MetE sequences (black pentagram). Based on this, *Schizochytrium* sp. can activate the expression of *MetE-like* for the function of MetE in a cobalamin-free environment.

To determine the effects of the AT and KS domains of *MetE-like* on the PKS pathway, we activated the expression of *MetE-like* by removing cobalamin from the *Schizochytrium* sp. culture medium. For the EPA ratio, considerable differences were observed with cobalamin addition (cobalamin^+^) and without cobalamin addition (cobalamin^−^) (Fig. 4c). Specifically, under cobalamin^−^, EPA proportion increased with the increase in fermentation time and finally reached 7.63%. In contrast, with cobalamin^+^, EPA proportion gradually decreased with the prolongation of the fermentation time and resulting in only 1.26% of EPA content. Finally, the EPA titer and EPA yield reached 335.9 mg/L and 15.2 mg/g cell dry weight, respectively, and were 2.03- and 3.89-folds that of Cobalamin^+^ (Supplementary Fig. S4). The DHA/n-6DPA ratio with cobalamin^−^ remained around 3.60 and was trending upwards, but the DHA/n-6DPA ratio under the cobalamin^+^ condition fluctuated around 3.0 (Fig. 4d). To further verify the relationship between PKS and *MetE-like*, we quantified the transcript levels of the four related domains by RT-PCR (Fig. 4e). Notably, *MetE-like* was the only gene with the increased transcript level in cobalamin^−^, which was 4.21-folds higher than that under the cobalamin^+^ condition. Intriguingly, under cobalamin^−^, *orf* A, *orf* B, and *orf* C transcript levels were reduced by 74.8%, 78.6%, and 85.4%, respectively. According to the genomic information, *Schizochytrium* sp. has only one MetE domain on *MetE-like*^40^. Due to the demand for Met, the transcription level of MetE was obviously increased; thus, the transcription levels of the AT and KS functional domains of *MetE-like* were also increased. These results support that *MetE-like* can regulate EPA synthesis via the PKS pathway.

Further, to limit *MetE-like* expression under the cobalamin^−^ condition, Met was selected as the MetE domain inhibitor. Moreover, Czco is a type of flavoprotein, which can use derivatives of riboflavin as a prosthetic group to participate in electron transfer^41^. Notably, adding Met or riboflavin to *Schizochytrium* sp. did not affect EPA accumulation under the cobalamin^+^ condition, and adding Met and riboflavin simultaneously slightly decreased EPA accumulation (Fig. 4f). Under cobalamin^−^, adding Met or adding Met and riboflavin decreased the ability of EPA accumulation, which means that Met can alleviate the malnutrition caused by the cobalamin^−^ condition; however, adding riboflavin alone has no effect. In cobalamin^+^, the DHA/n-6DPA ratio is basically stable under the three additional conditions, meaning that the PKS system was stable. However, in cobalamin^−^, the DHA/n-6DPA ratio was similar to that of EPA, and Met could ameliorate the disorder of PKS system (Fig. 4g).

Further, the *MetE-like* transcript levels in *Schizochytrium* sp. under different fermentation conditions were tested. In cobalamin^+^, all inhibitor addition regimens caused a little difference (Fig. 4h). In contrast, under cobalamin^−^, the *MetE-like* transcript levels were reduced by 36.2%, 1.8%, and 49.5%, respectively. This means that the requirement for MetE function in *Schizochytrium* sp. is the direct driving force for the increase of *MetE-like* transcription levels under the cobalamin^−^ condition. Collectively, by regulating *MetE-like* transcription levels, the ability of *Schizochytrium* sp. to accumulate EPA and DHA/n-6DPA in the PKS pathway show corresponding changes; hence, *MetE-like* can regulate the synthesis of EPA via the PKS pathway.

### Exploring the lipid compound composition under the condition of *MetE-like* activation

**Fig. 5:**
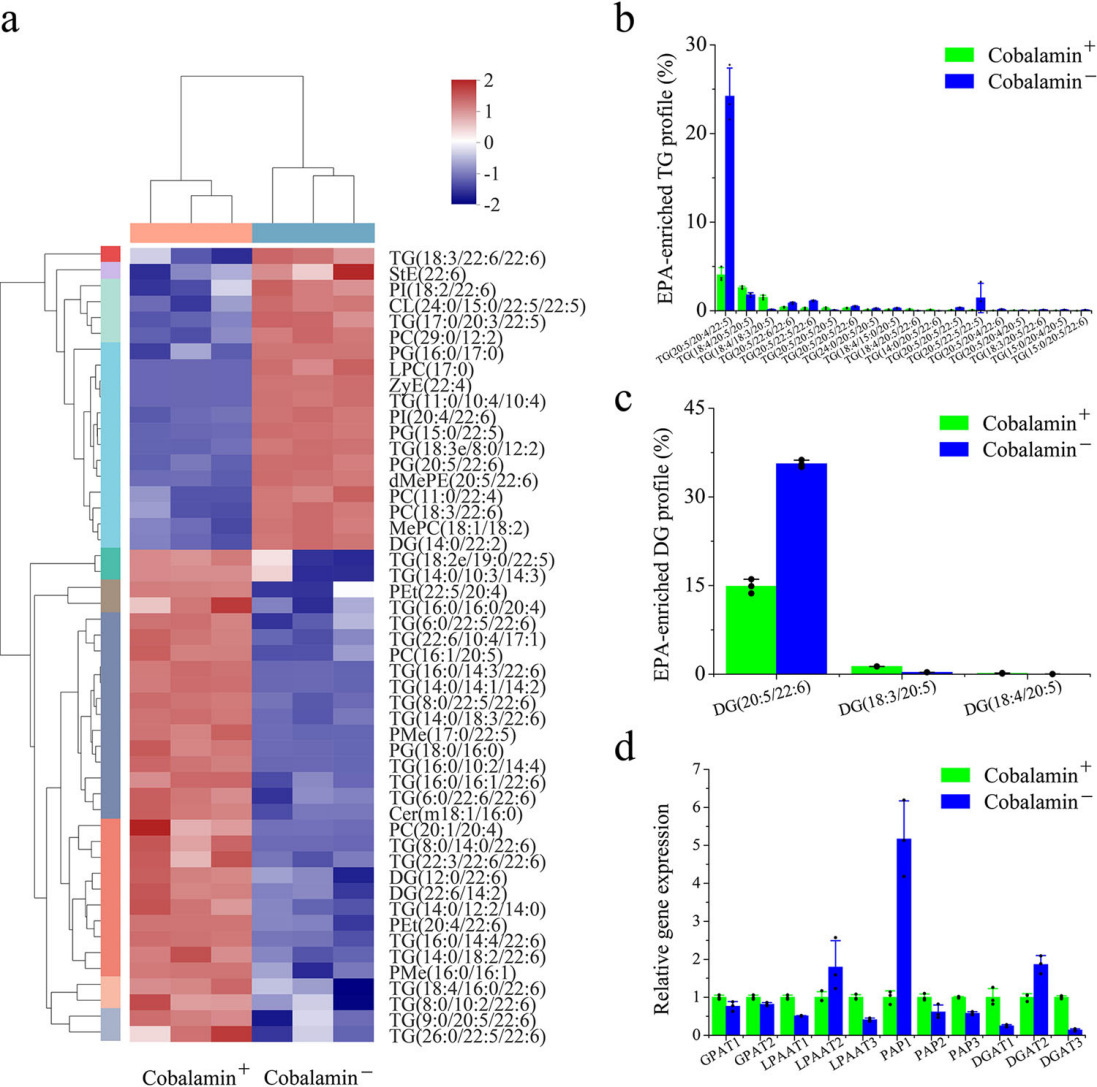
Differences in lipid compound composition in *Schizochytrium* sp. under *MetE-like* activation. **a** Lipid compounds cluster analysis plot. The colors in the figure represent the relative expression levels of lipid compounds in this group of samples, the left side is the dendrogram of lipid compound clustering, and the right side is the name of the lipid compounds. **b**, **c** EPA-enriched TG profile (**b**) and EPA-enriched DG profile (**c**) in *Schizochytrium* sp.(60 h). **d** Comparison of fatty acid storage related genes transcript level between cobalamin^+^ and cobalamin^−^ of *Schizochytrium* sp. (60 h).

To further investigate the effect of cobalamin on EPA accumulation in *Schizochytrium* sp., lipidomics analysis was performed to analyze the differences in lipid compound compositions during the late stage of *Schizochytrium* sp. fermentation under cobalamin^+^ and cobalamin^−^ conditions. Metabolites with similar expression patterns are often functionally related, and a total of 50 metabolites from the metabolic set were selected for the cluster analysis (Fig. 5a). Among them, 25 lipids were triglycerides (TGs). TG is the major storage form of fatty acids in *Schizochytrium* sp.^15^. In addition, fatty acids in lipid storage forms such as phosphatidylcholine (PC), diradylglycerols (DG), phosphatidylglycerol (PG), lysophosphatidylcholine (LPC), and dimethylphosphatidylethanolamine (dMePE) were also obviously altered. DHA is a characteristic fatty acid of *Schizochytrium* sp.^34^, and 25 lipid compounds showed the variability in DHA storage. A few lipids contain EPA, and only PC (16:1/20:5), TG (9:0/20:5/22:6), PG (20:5/22:6), and dMePE (20:5/22:6) majorly contribute to the variability in EPA storage. In the cobalamin^−^ condition, LPC (17:0), PG (16:0/17:0), PG (15:0/22:5), and TG (17:0/20:3/22:5) were increased, whereas TG (16:0/16:0/20:4), TG (16:0/14:3/22:6), TG (14:0/14:1/14:2), PG (18:0/16:0), and TG (18:4/16:0/22:6) were decreased.

Next, we analyzed the differences among the lipids registered in HMDB. Generally, fatty acids in *Schizochytrium* sp. are mainly composed of glycerolipids (GLs) and glycerophospholipids (GPs)^42^. GLs and GPs accounted for more than 95% of the total lipids in lipids registered in HMDB; however, under cobalamin^−^, GL proportion increased by 9.9%, whereas GP proportion decreased by 13.4% (Supplementary Fig. S5). Among GLs, under cobalamin^+^, the proportion of TG to GLs was about 70%, whereas TG only accounted for 54.8% of GLs under the cobalamin^−^ condition; however, under cobalamin^−^, DG proportion increased by 60% (Supplementary Fig. S6). PC is the main storage form of GPs and accounts for more than 90%; phosphatidylethanolamine (PE) is the second-largest storage form of GPs, but PE proportion increased by 86.5% under the cobalamin^−^ condition (Supplementary Fig. S7).

Furthermore, we analyzed the differences in EPA content among the lipids registered in HMDB. In EPA-containing TG, its proportion (20:5/20:4/20:5) increased and up to 23.99% under the cobalamin^−^ condition, which was about 6.8 times the proportion under the cobalamin^+^ condition. A total of 18 EPA-containing TGs were registered in HMDB, of which 13 were obviously increased (Fig. 5b). Similarly, a total of three EPA-containing DGs were registered in HMDB, of which the DG proportion (20:5/22:6) in the cobalamin^−^ condition was 35.63%, whereas that in the cobalamin^+^ condition was 14.82%, but the proportions of the other two EPA-containing DGs were less (Fig. 5c). In addition, among GPs registered in HMDB, PE, glycerophosphates (PA), and PC became the main EPA-storage forms. In cobalamin^−^, the proportion of PE (20:5/22:6) increased from 2.12% to 15.53%, while the proportion of EPA in PCs has generally increased, but the proportion of PA (20:5/22:6) has decreased (Supplementary Figs. S8–S10). Interestingly, the co-storage form of EPA and DHA was preferred among diester lipids. *Schizochytrium* sp. tended to store EPA in GLs form under the cobalamin^−^ condition.

To determine the changes that occur in fatty acid storage in *Schizochytrium* sp., we quantified the transcript levels of glycerol-sn-3-phosphate acyl-transferase (GPAT), lysophosphatidate acyl-transferase (LPAAT), phosphatidic acid phosphatase (PAP), and diacylglycerol acyltransferase (DGAT) genes under the cobalamin^+^ and cobalamin^−^ conditions by RT-PCR. TGs are synthesized via the Kennedy pathway in *Schizochytrium* sp.^15^. In the first step, glycerol-3-phosphate is converted into lysophosphatidate (LPA) by the rate-limiting enzyme GPAT. Subsequently, LPAAT transfers an acyl moiety from coenzyme A (CoA) onto the sn-2 position of LPA, resulting in PA. Then, PAP catalyzes the formation of DG from PA, and finally, DGAT is catalyzed to transfer an acyl group from CoA to DG to form TG. Although the overall transcript levels of related genes decreased obviously, the transcript levels of LPAAT2, PAP1, and DGAT2 increased 0.80, 4.17, and 0.86 times, respectively, which may be an important reason for the difference in fatty acid composition in *Schizochytrium* sp. (Fig. 5d).

### Relieving the growth stress to increase the peak production of EPA by optimizing carbon sources

**Fig. 6:**
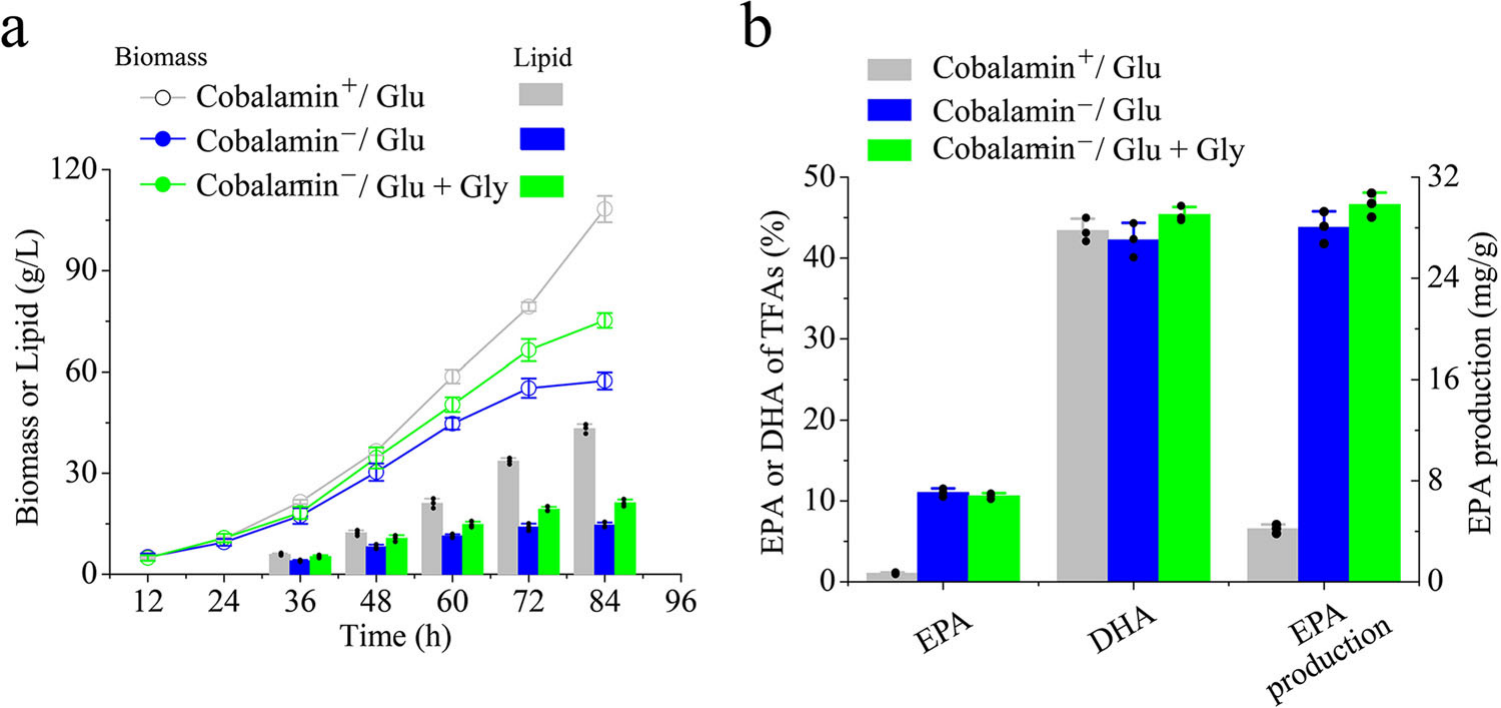
Production of EPA using *Schizochytrium* sp. by fed-batch culture. **a** Time courses of Biomass (or dry cell weight) and Lipid in fed-batch culture. **b** EPA ratio, DHA ratio, and EPA production (mg/g dry cell weight) of *Schizochytrium* sp. (84 h) in fed-batch culture. The statistical significances of the final results were analyzed by t-test, *p < 0.05.

To explore the effect of cobalamin on the EPA production capacity of *Schizochytrium* sp., a mixed carbon source of crude glycerol and glucose was used to relieve the growth stress on *Schizochytrium* sp. under the cobalamin^−^ condition^43–45^. Here, to explore the effect of cobalamin on the EPA production capacity of *Schizochytrium* sp., a mixed carbon source of crude glycerol and glucose was used to relieve the growth stress on *Schizochytrium* sp. under the cobalamin^−^ condition. In the pre-experiment, we tested the changes caused by the different ratio of carbon sources under the cobalamin^−^ condition and the final mixed carbon source of 20 g/L crude glycerol + 40 g/L glucose was selected (Supplementary Fig. S11). As shown in Fig. 6a, under cobalamin^−^, the final biomass and lipids via fed-batch fermentation were far lower than under the cobalamin^+^ condition, but the mixed carbon source was favorable for biomass and lipid accumulation, which finally reached 75.13 g/L and 21.23 g/L, respectively. However, mixed carbon source cannot fundamentally solve the growth problem caused by cobalamin deficiency.

Unexpectedly, carbon source optimization did not increase EPA proportion, and EPA proportion obtained using the sole glucose and mixed carbon source reached 11.1% and 10.6%, respectively, whereas it was only about 1% under the original fermentation conditions. No obvious difference in DHA proportions among the three fermentation conditions was observed; however, under cobalamin^−^, the use of mixed carbon sources improved DHA accumulation, and DHA accounted for 45.4%. In addition, studies indicated that the DHA content was enhanced when glycerol was used as carbon source, and subsequent transcriptomic analysis revealed that the glycolysis and amino acid metabolism were greatly upregulated^46^. Finally, under cobalamin^−^, the peak production of EPA obtained by using mixed carbon sources reached 29.83 (mg/g cell dry weight), which was 7.12-folds higher than that of the original fermentation conditions (Fig. 6b). Therefore, we have established a plausible method for the co-production of EPA and DHA in *Schizochytrium* sp. by fermentation engineering.

### Conclusion

The *Schizochytrium* sp. was selected as the potential EPA produce by analyzing the fatty composition of different thraustochytrids. We first identified the *MetE-like* complex that can modulate the PKS system to produce EPA. When the transcriptional level of *MetE-like* was activated, the production of EPA can be increased by 7.12-folds compared to that of the original fermentation condition, resulting a co-production of 2.25 g/L EPA and 9.59 g/L DHA. The results of the present study provide important insights into the role of PKS system for producing fatty acids and report the ability of *Schizochytrium* sp. As a potential EPA and DHA co-producer (Table 1).

**Table 1:** The ability of microorganisms to co-produce EPA and DHA.

Strain	EPA of TFA (%)	EPA (g/L)	DHA of TFA (%)	DHA (g/L)	Reference
*Hondae*a gen. nov.	0.41 ± 0.01	0.01 ± 0.01	50.12 ± 1.23	0.06 ± 0.01	^9^
*Parietichytrium* sp.	2.11 ± 0.17	0.08 ± 0.01	5.1 ± 0.3	0.19 ± 0.02	^10^
*Schizochytrium* sp. A-2	2.01 ± 0.20	0.13 ± 0.01	53.2 ± 0.30	3.44 ± 0.02	^42^
*Nannochloropsis oceanica*	4.12 ± 0.35	0.02 ± 0.01	2.37 ± 0.40	0.01 ± 0.01	^47^
*Phaeodactylum tricornutum* and *Aurantiochytrium limacinum*	17.96 ± 1.23	0.25 ± 0.01	18.42 ± 0.45	0.26 ± 0.01	^48^
*Rhodomonas baltica* NIVA-5/91	12.1 ± 1.07	0.88 ± 0.07	12.8 ± 0.28	0.95 ± 0.03	^49^
*Phaeodactylum tricornutum* CCMP 2561	26.2 ± 0.36	1.64 ± 0.05	4.93 ± 0.09	0.25 ± 0.02	^49^
*Schizochytrium* sp. HX-308	10.6 ± 0.34	2.25 ± 0.24	45.4 ± 0.95	9.59 ± 0.20	This study

## Methods

### Strains, media, and growth conditions

*Schizochytrium* sp. CCTCC M 209059, *Hondaea* sp., and *Aurantiochytrium* sp. were isolated from sea water and stored in the China Center for Type Culture Collection. *Thraustochytrium* sp. ATCC 26185 was obtained from the American Type Culture Collection (USA). Thraustochytrid strains were grown in 50 mL of seed culture medium [40 g/L glucose, 10 g/L sodium glutamate, and 4 g/L yeast extract in artificial sea water (1.5 g/L NaCl, 0.7 g/L KCl, 4.8 g/L MgSO_4_·7H_2_O, 0.1 g/L CaCl_2_·2H_2_O, 10 g/L Na_2_SO_4_)] with 0.25 mg/L vitamin B12 and 1 g/L trace elements (6 mg/L Na_2_EDTA, 8 mg/L MnCl_2_·4H_2_O, 0.8 mg/L ZnSO_4_, 0.29 mg/L FeSO_4_, 0.01 mg/L CoCl_2_·6H_2_O, 0.6 mg/L CuSO_4_·5H_2_O, 0.06 mg/L NiSO_4_·6H_2_O, and 0.1 mg/L Na_2_MoO_4_·2H_2_O) in a 250 mL flask at 28 °C with rotation at 170 rpm. The fermentation medium contained 50 g/L glucose, 10 g/L yeast extract, and 20 g/L sodium glutamate, the rest of the ingredients are the same with seed culture medium. Cells were cultured in the seed culture medium for three generations, and then 10 mL of the culture was transferred to the 90 mL fermentation medium in 500 mL flasks at 28 °C with shaking at 170 rpm.

### Glucose content, biomass, lipid, and fatty acids analysis

Fresh cells were collected and diluted it 100-fold, then centrifuged to take the supernatant, and glucose concentration was determined by a bioanalyzer (SBA-40ES, YanHe Biotechnology, China). The Biomass (dry cell weight) was determined gravimetrically by filtering 10 mL of the samples using a centrifuge for 5 min at 6500 × *g*. The cells were transferred to dried and weighed filter paper, dried at 60 °C. During the drying process, the weight was recorded repeatedly until the mass remained stable. The fermentation broth (50 mL) was added to a high-pressure homogenizer to crush *Schizochytrium* sp. HX-308 cells, then the lipids were extracted with an equal volume of n-hexane for three consecutive times, and a rotary vacuum evaporator was used to remove n-hexane at 45 °C. Finally, the total lipids were calculated by weighing. Fatty acid composition was calculated by the percentage of fatty acid methyl esters (FAMEs) in total fatty acids (TFAs).

### RT-PCR analysis

*Schizochytrium* sp. was cultured at 28 °C with shaking at 170 rpm, cells (20 mL) were harvested at 12,000 × *g* and 4 °C for 5 min. The *Schizochytrium* sp. cells were milled with liquid nitrogen, and 50 mg of them were taken for RNA extraction. Total RNA was isolated using FastPure Universal Plant Total RNA Isolation Kit (Vazyme, China), and synthesis of cDNA was carried out with HiScript III RT SuperMix for qPCR (+gDNA wiper) (Vazyme, China). Then, the cDNA was used as a template to perform RT-PCR with 2 × Vazyme LAmp Master Mix (Vazyme, China), the primers listed in Supplementary Table 1.

### Transcriptomic and lipidomics analysis

*Schizochytrium* sp. cells (50 mL) were harvested at 10,000 × *g* and 4 °C for 10 min, and immediately quenched with liquid nitrogen, then cells were stored at −80 °C. High-resolution genome-wide transcription analysis was performed using RNA‐Seq at Shanghai Majorbio Bio-pharm Technology Co., Ltd (Shanghai, China). Raw reads were filtered to remove adapters and low-quality reads, and the remaining reads were mapped to the reference genome for transcript information. Gene expression was normalized by transcripts per kilobase per million mapped reads (TPM).

Likewise, the lipid sample is separated by liquid chromatography, the single component is then ionized into the ion source of the high vacuum mass spectrometer, separated by the mass-to-charge ratio (*m*/*z*) to obtain the mass spectrometry map, and finally through the mass spectrometry data analysis of the sample, the qualitative quantitative results of the sample are obtained. The data were analyzed on the online platform of Majorbio Cloud Platform (www.majorbio.com). All metabolites identified by mass spectrometry were compared with the HMDB 4.0 (Human Metabolome Database, www.hmdb.ca) database, and the annotation information of the metabolites in the database was obtained.

### Statistics and reproducibility

Each experiment was carried out in three biological replicates, with results presented as mean ± standard deviation (SD). Statistical analysis was performed using Graphpad Prism 5 (GraphPad, USA). Letters above the bars indicate significant difference (*p* ≤ 0.05), based on one-way analysis of variance (ANOVA) and Tukey’s honestly significant difference (HSD) test. The statistical significances of the final results were analyzed by t-test, *p < 0.05. Genome sequence data of *Schizochytrium* sp. CCTCC M 209059 has been deposited in the National Center for Biotechnology Information (NCBI) GenBank database.

### Reporting summary

Further information on research design is available in the Nature Portfolio Reporting Summary linked to this article.

## Supplementary information

Supplementary Information

Description of Additional Supplementary Files

Supplementary Data 1

Supplementary Data 2

Supplementary Data 3

Supplementary Data 4

Supplementary Data 5

Supplementary Data 6

Supplementary Data 7

Supplementary Data 8

Supplementary Data 9

Supplementary Data 10

Supplementary Data 11

Supplementary Data 12

Reporting Summary

## Data Availability

The authors declare that all data supporting the findings of this study are available within the article and its supplementary information (Figs. S1–S11 and Table S1). Source data used to generate Supplementary Figs. 1–11 are available as Supplementary Data 1–11, and source data used to generate Figs. 1–6 are available as Supplementary Data 12. *Schizochytrium* sp. CCTCC M 209059 (GenBank accession no. KN805382.1) and *MetE-like* (Gene ID A0661) data from the corresponding author upon reasonable request.
