# Supplementary material for: Efficient co-production of EPA and DHA by Schizochytrium sp. via regulation of the polyketide synthase pathway
Source: Commun Biol. 2022 Dec 9;5:1356. doi: 10.1038/s42003-022-04334-4 (PMC9734096; doi:10.1038/s42003-022-04334-4)
Supplement: Supplementary file 1 — Supplementary Information [file 42003_2022_4334_MOESM1_ESM.pdf]

Efficient co-production of EPA and DHA by *Schizochytrium* sp. via regulation of the polyketide synthase pathway

Wang Ma[1,2], Meng-Zhen Liu[1], Zi-Xu Zhang[1], Ying-Shuang Xu[1], Peng-Wei Huang[1,2], Dong-Sheng Guo[1], Xiao-Man Sun[1]*, He Huang[1,3]*

[1]School of Food Science and Pharmaceutical Engineering, Nanjing Normal University, 2 Xuelin Road, Qixia District, Nanjing, China

[2]College of Life Sciences, Nanjing Normal University, 2 Xuelin Road, Qixia District, Nanjing, China

[3]College of Biotechnology and Pharmaceutical Engineering, Nanjing Tech University, No. 30 South Puzhu Road, Nanjing, China

*Correspondence: xiaomansun@njnu.edu.cn; huangh@njnu.edu.cn

This PDF file includes:

Tables S1

Figures S1 to S11

Supplementary Table1. Primers used in this study.

Target gene	Primer	Sequence (5′–3′)
β-actin	F	TGTCCTCACGCTCAAGTA
	R	GAAGGTGTGGTGCCAGAT
△9 elongase	F	TCCTCTCATTACGCAGTTG
	R	TTCACTATGACTGCGAGC
△6 desaturase	F	CGAGCATACAGTTGACAT
	R	AATTGCCAGGTGATTCAT
△8 desaturase	F	CGCATTCCTTATATTGTTCT
	R	AACACTGACAACTGGATT
△12 desaturase	F	ACTTTCTCCCTTACCTTGT
	R	CATACTCACCAAGATGTCC
MetE-like	F	TACATTGACTACATTAACTTCGT
	R	GTTCTTGCTGTTGTGAGT
orf A	F	CATCAAGGAGAACGAGAC
	R	AGGGAATTTGAACTGCTC
orf B	F	GAAGAAGAAGAAGAAGAAGAAGAA
	R	AGATCCGATCCTCAACAAG
orf C	F	TCTTGAGACCGTTAATGTAA
	R	CATCGAGAAGCGTATCTT
GPAT1	F	ACCGACAAGAATGGAGAA
	R	AGATGAAGATGCTGTTGC
GPAT2	F	AACAAGAAGTCAAGAACAT
	R	CGTAGATAGACTGCGTAT
LPAAT1	F	ATTGATTACGAAGAGTTCT
	R	CTCAGGAACCATTAACTT
LPAAT2	F	TTACCTTGATCTGATTGA
	R	CTTAACGAGAATATGCTC
LPAAT3	F	AAACCCACACCGACAAGA
	R	CAATCCAACTTCCTCATCTTCATA
PAP1	F	CTTGGAGTATCACAGTGT
	R	GGATGGTTCTTCGTATGG
PAP2	F	ATTTCCAGAACCCGATAT
	R	AATCAAGTTATCCTCGGT
PAP3	F	CAATTTGACCGCTACTAT
	R	CCATTATCCTTCTCTTGC
DGAT1	F	TTCCTCTCGCCAACATAA
	R	AGAAATGGAAATCACTTGGTT
DGAT2	F	CAAAGACCACAGCACATC
	R	ATTGTGCTAGGTGTTCTCT
DGAT3	F	GTGATCCGTACATTGCTA
	R	GTTAATACGATGACCTCTTG

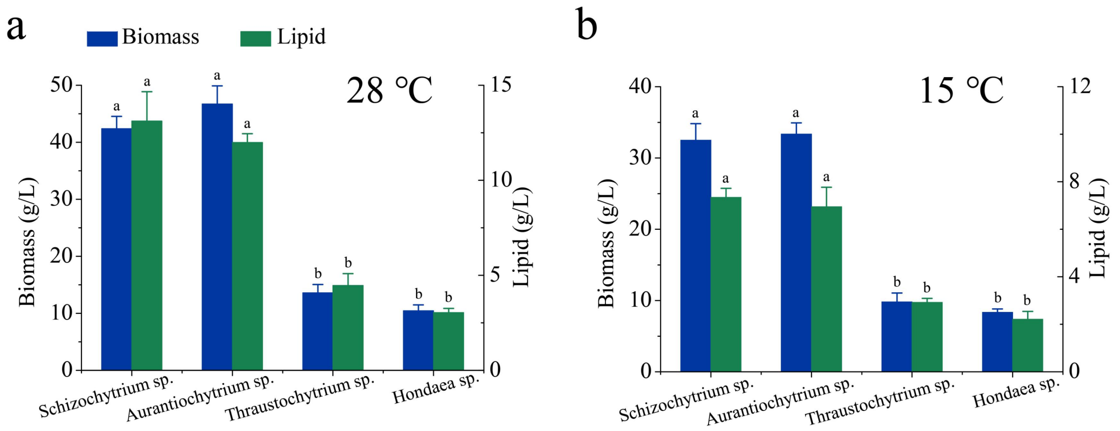

Supplementary Figure 1. Biomass and lipid accumulation of different thraustochytrid genera under (a) 28°C fermentation conditions and (b) 15 °C fermentation conditions. Letters above the bars indicate significant difference ($p \leq 0.05$), based on one-way analysis of variance (ANOVA) and Tukey's honestly significant difference (HSD) test. The data shown are the mean ± S.D. (n=3).

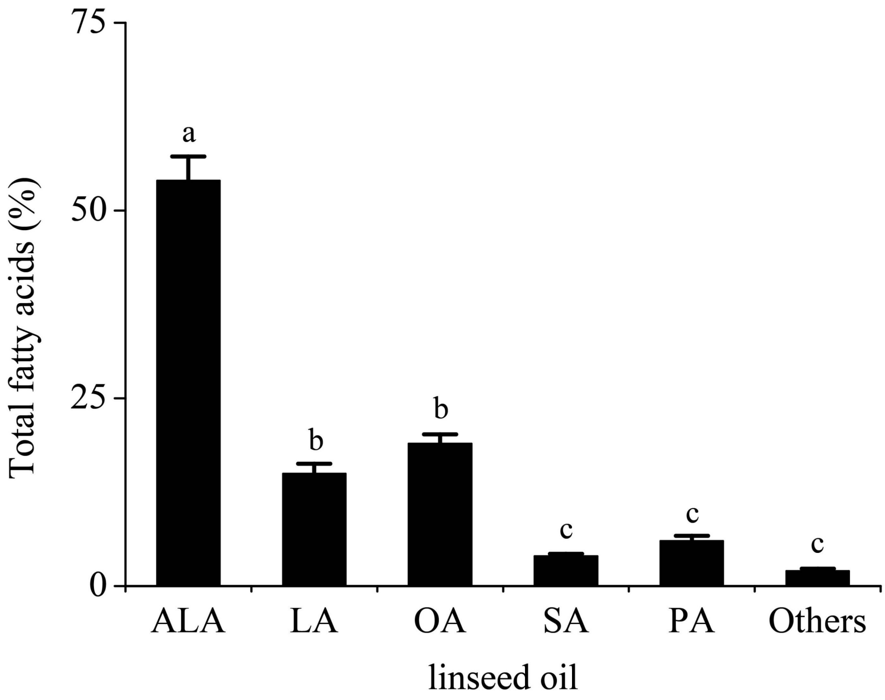

Supplementary Figure 2. Fatty acid composition of linseed oil. ALA α-linolenic acid, LA linoleic acid, OA oleic acid, SA stearic acid, PA palmitic acid. Letters above the bars indicate significant difference ($p \leq 0.05$), based on one-way analysis of variance (ANOVA) and Tukey's honestly significant difference (HSD) test. The data shown are the mean ± S.D. (n=3).

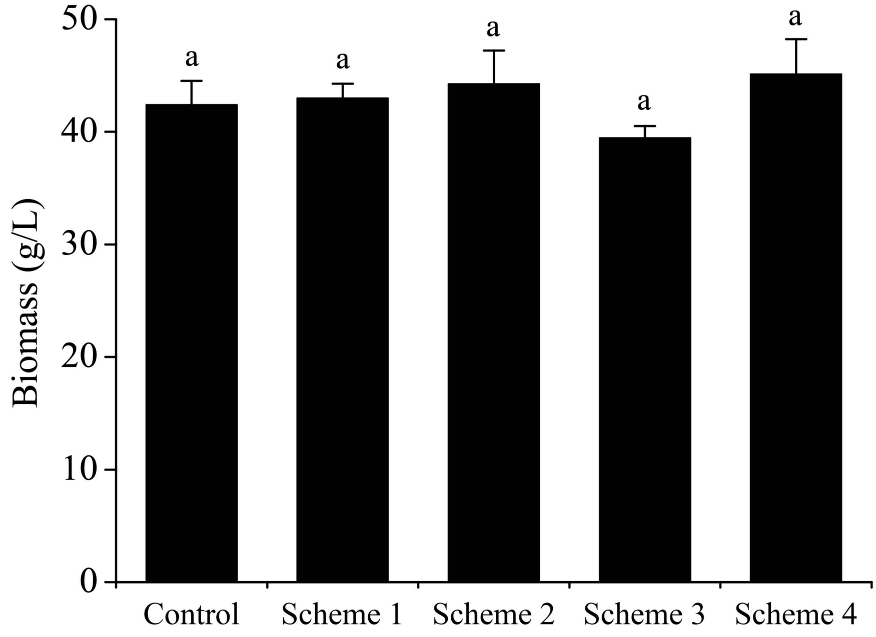

Supplementary Figure 3. Biomass of Schizochytrium under different adding schemes. Letters above the bars indicate significant difference ($p \leq 0.05$), based on one-way analysis of variance (ANOVA) and Tukey's honestly significant difference (HSD) test. The data shown are the mean ± S.D. (n=3).

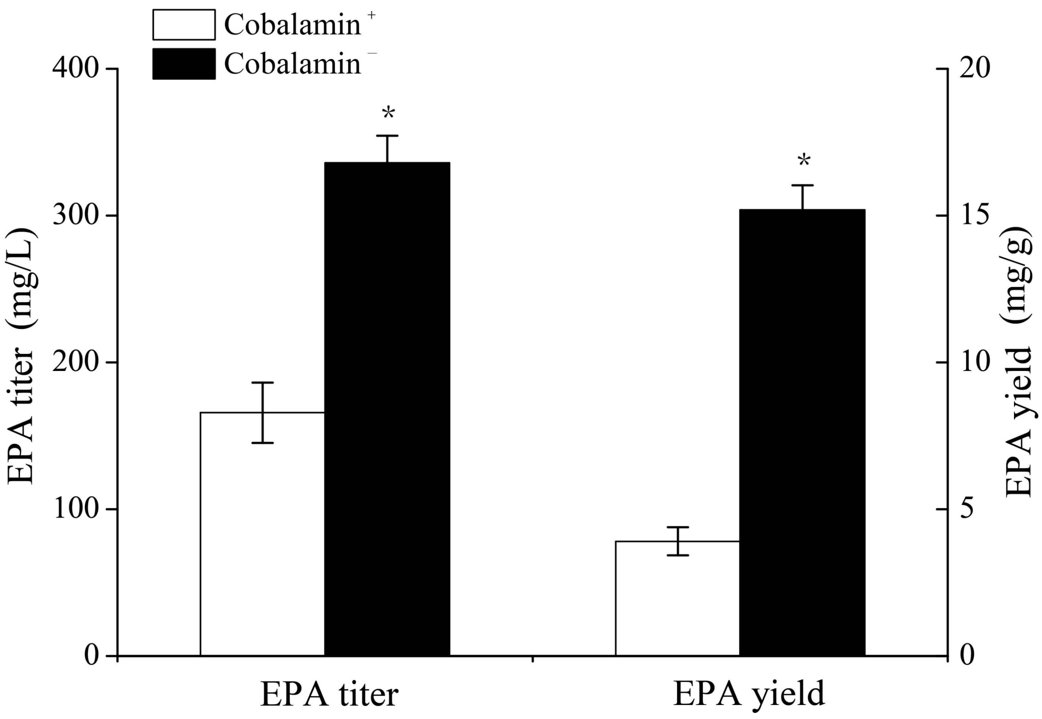

Supplementary Figure 4. Effects of EPA yield and EPA titer between cobalamin^{+} and cobalamin^{-} of *Schizochytrium* sp.. The statistical significances of the final results were analyzed by t-test, *p < 0.05. The data shown are the mean ± S.D. (n=3).

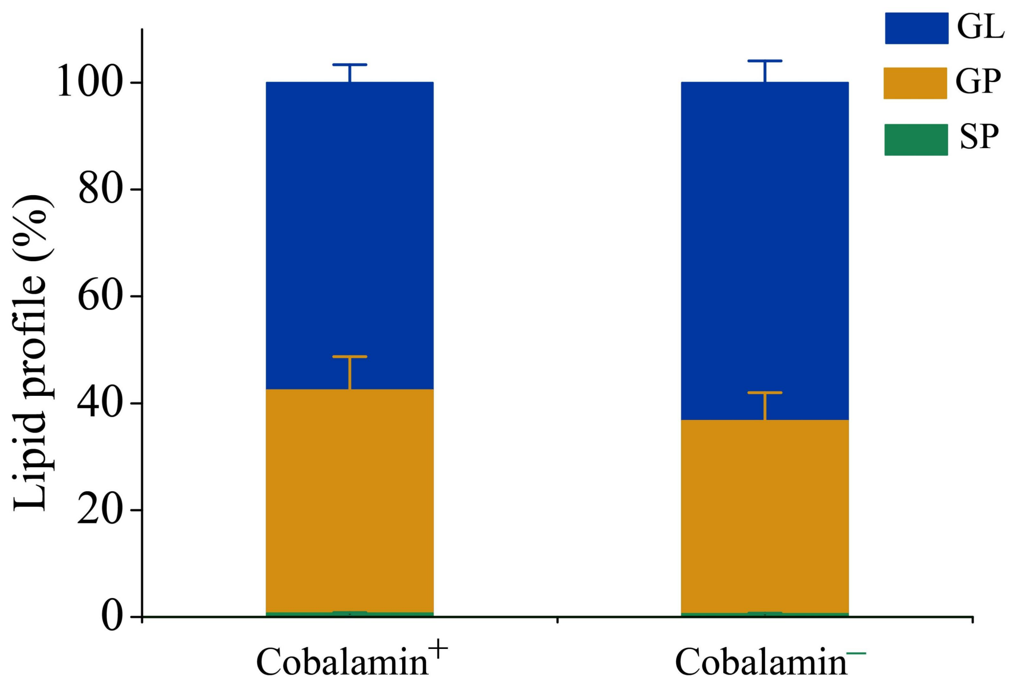

Supplementary Figure 5. Distribution of fatty acid storage in *Schizochytrium* sp. under different fermentation conditions. GL Glycerolipids, GP Glycerophospholipids, SP Sphingolipids. The data shown are the mean ± S.D. (n=3).

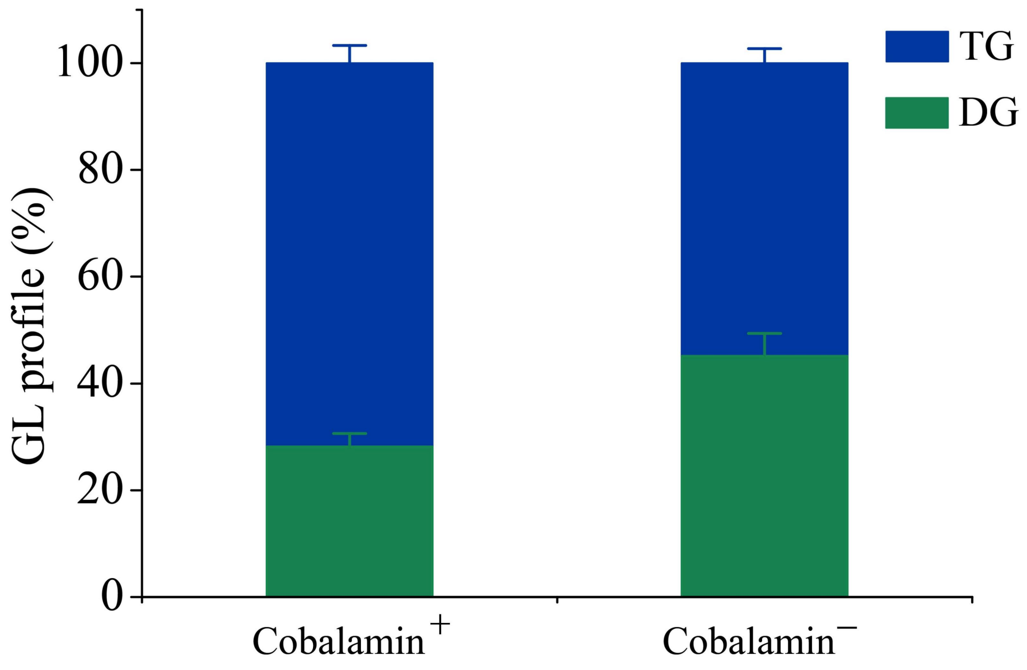

Supplementary Figure 6. Glycerolipid composition of *Schizochytrium* sp.. TG Triradylcglycerols, DG Diradylglycerols. The data shown are the mean ± S.D. (n=3).

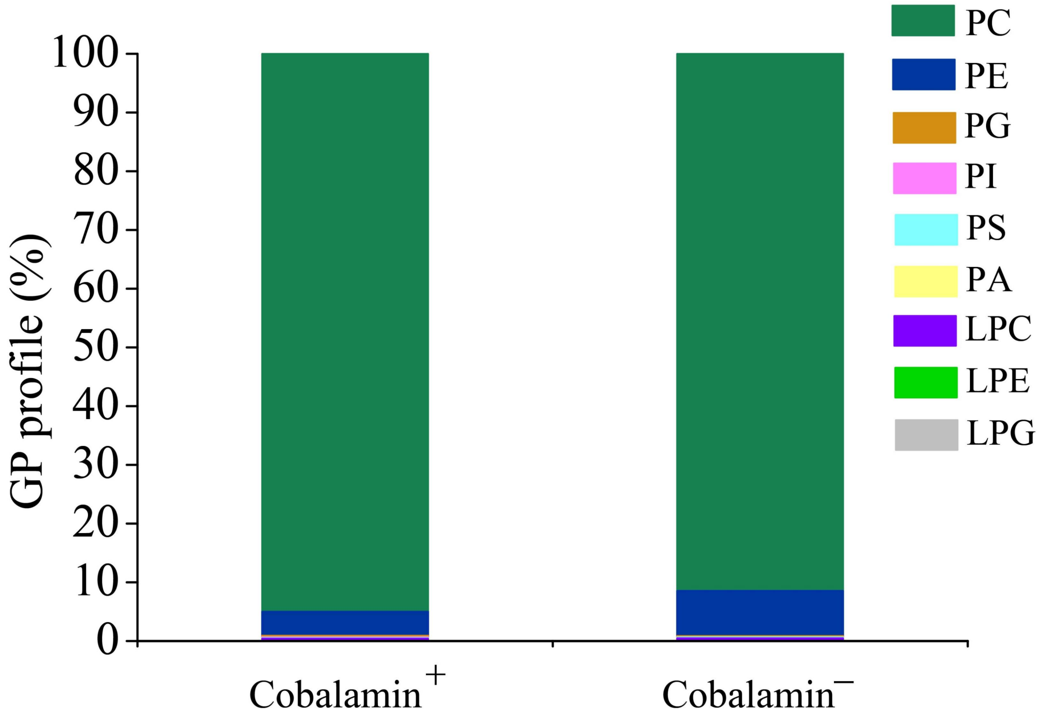

Supplementary Figure 7. Glycerophospholipid composition of *Schizochytrium* sp..

PC phosphocholines, PE phosphoethanolamines, PG phosphoglycerols, PI phosphoinositols, PS phosphoserines, PA phosphates, LPC lysophosphatidylcholine, LPE lysophosphatidylethanolamine, LPG lysophosphatidylglycerol. The statistical significances of the final results were analyzed by t-test, *$p < 0.05$.

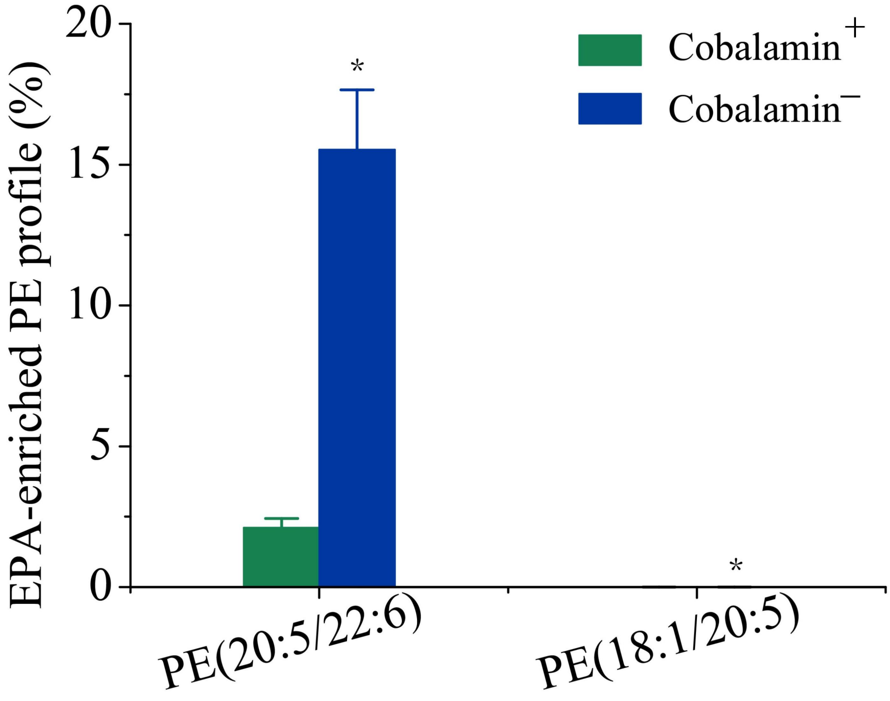

Supplementary Figure 8. EPA-enriched PE profile in *Schizochytrium* sp.(60 h). The statistical significances of the final results were analyzed by t-test, *$p < 0.05$. The data shown are the mean ± S.D. (n=3).

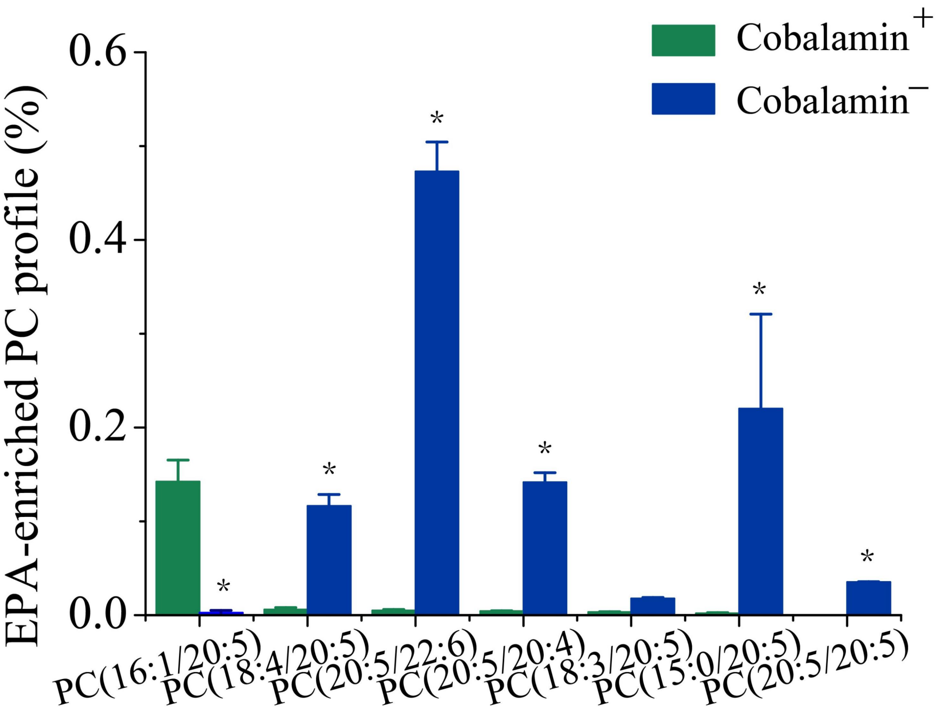

Supplementary Figure 9. EPA-enriched PC profile in *Schizochytrium* sp.(60 h). The statistical significances of the final results were analyzed by t-test, *$p < 0.05$. The data shown are the mean ± S.D. (n=3).

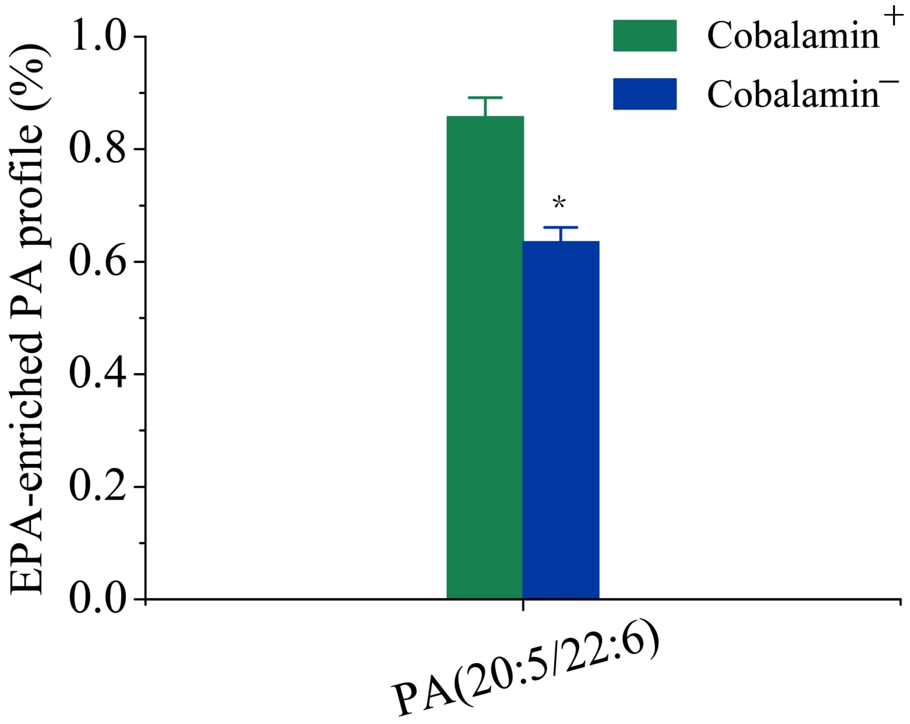

Supplementary Figure 10. EPA-enriched PA profile in *Schizochytrium* sp.(60 h).

The statistical significances of the final results were analyzed by t-test, *$p < 0.05$. The data shown are the mean ± S.D. (n=3).

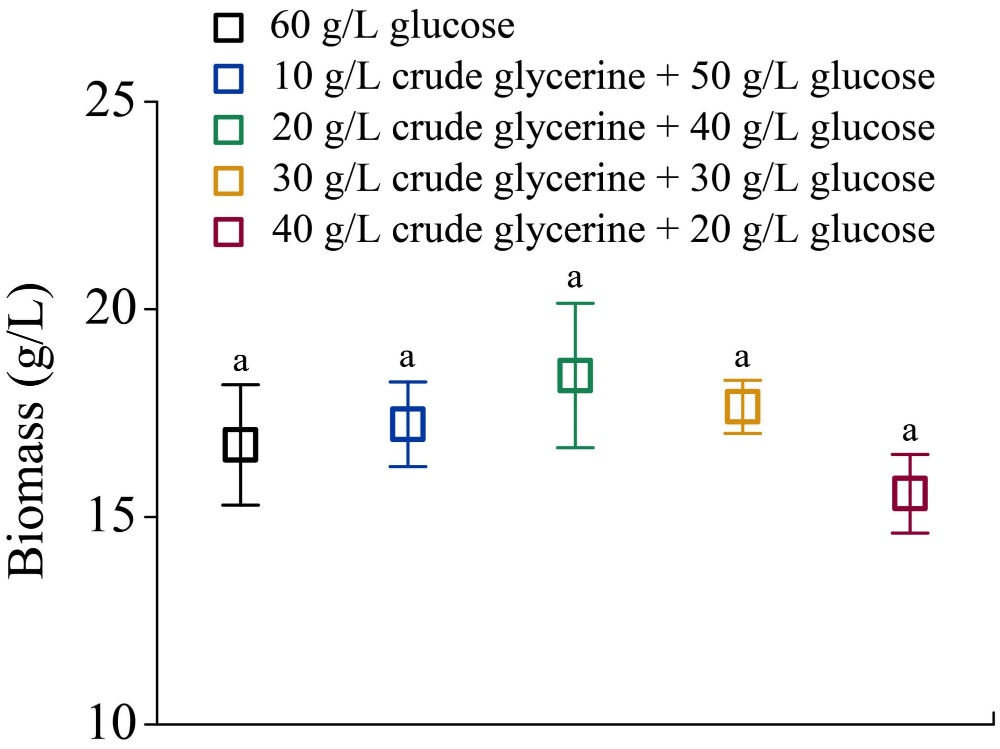

Supplementary Figure 11. Effects of mixed carbon sources of different proportions of crude glycerol and glucose on the Biomass of *Schizochytrium* sp.. Letters above the bars indicate significant difference ($p \leq 0.05$), based on one-way analysis of variance (ANOVA) and Tukey's honestly significant difference (HSD) test. The data shown are the mean ± S.D. (n=3).
