# Supplementary material for: Efficient co-production of EPA and DHA by Schizochytrium sp. via regulation of the polyketide synthase pathway
Source: Commun Biol. 2022 Dec 9;5:1356. doi: 10.1038/s42003-022-04334-4 (PMC9734096; doi:10.1038/s42003-022-04334-4)
Supplement: Supplementary file 2 — Description of Additional Supplementary Files [file 42003_2022_4334_MOESM2_ESM.pdf]

Description of Additional Supplementary Files

File name: Supplementary Data 1-11

Description: Source data used to generate Supplementary figure 1-11.

File name: Supplementary Data 2

Description: source data used to generate figure 1-6.
